# Gastric neuroendocrine tumors: a comprehensive analysis of clinicopathological characteristics and survival outcomes from a reference center

**DOI:** 10.1590/0102-67202025000056e1925

**Published:** 2026-04-10

**Authors:** Mateus Barradas RIBEIRO, Marina Alessandra PEREIRA, Donato Roberto MUCERINO, Osmar Kenji YAGI, André Roncon DIAS, Bruna de Camargo NIGRO, Ulysses RIBEIRO, Marcus Fernando Kodama Pertille RAMOS

**Affiliations:** 1 Universidade de São Paulo, Faculty of de Medicine, Instituto do Cancer do Estado de São Paulo, Department of Gastroenterology – São Paulo (SP), Brazil.

**Keywords:** Stomach neoplasms, Neuroendocrine tumors, Gastrectomy, Survival analysis, Endoscopy, Neoplasias Gástricas, Tumores Neuroendócrinos, Gastrectomia, Análise de Sobrevida, Endoscopia

## Abstract

**Background:**

Gastric neuroendocrine tumors (gNETs) are uncommon neoplasms arising from enterochromaffin-like cells, representing a distinct subset of gastric malignancies, with challenging clinical management.

**Aims:**

To analyse the classification, treatment indication, and survival of patients diagnosed with gNETs.

**Methods:**

We retrospectively analyzed patients diagnosed with gNETs between 2009 and 2025 at a high-volume tertiary center in Brazil. Clinical, pathological, and treatment data were reviewed, and tumors were classified according to World Health Organization and clinicopathological criteria into Types I, II, and III.

**Results:**

Of the 75 patients included, 53 (70.7%) were classified as Type I, 5 (6.7%) as Type II, and 17 (22.6%) as Type III. Treatment included surgery in 25 patients (33.3%) and endoscopic resection in 50 (66.7%). Type I tumors predominated in females (p<0.001), were frequently multifocal (p<0.001), associated with higher body mass index (p=0.002), and were mainly managed endoscopically (p=0.008). Type II tumors were rare and associated with multiple endocrine neoplasia Type 1, while Type III tumors were predominantly male, larger, high-grade (G3), and frequently metastatic, requiring surgical resection and palliative therapy. Among the 25 surgically treated patients, most were men (52%) and included 12 patients (48.0%) with Type I, 3 (12.0%) with Type II, and 10 (40.0%) with Type III tumors. Survival analysis showed significantly worse outcomes for Type III and G3 tumors. Multivariable analysis identified advanced age (hazards ratio 4.11; 95% confidence interval (95%CI): 1.14–14.80; p=0.030) and tumor, lymph node, metastasis (TNM) stage III/IV (HR 5.42; 95%CI: 1.26–23.26; p=0.023) as independent predictors of poorer survival.

**Conclusions:**

gNETs exhibit heterogeneous clinical behavior, with Type I tumors predominating in the Brazilian population. Tumor type, grade, and TNM stage are critical determinants of prognosis and should guide individualized treatment strategies.

## ARTICLE HIGHLIGHTS

In one of the largest Brazilian gNET series, Type I tumors predominated, showing an indolent course and endoscopic management, while Type III was aggressive, high-grade, and frequently metastatic.

Advanced age and TNM stage III/IV were identified as the strongest independent predictors of poorer overall survival, highlighting the importance of early detection.

Our findings reinforce that accurate tumor classification by type and grade is essential to guide individualized treatment strategies, optimizing the prognosis for gNET patients.

## Summary

**CENTRAL MESSAGE:**

The stomach is the most frequent site of gastrointestinal neuroendocrine neoplasms (NENs), with an annual incidence of approximately 0.4 per 1,00,000 individuals. According to the most recent World Health Organization Classification of Tumors of the Digestive System, NENs are divided into three major categories: gastric neuroendocrine tumors, which are the most common, well-differentiated, and have any grade; neuroendocrine carcinomas, which are poorly differentiated and high-grade; and mixed neuroendocrine-non-neuroendocrine neoplasms, which are aggressive and harbor multiple molecular alterations. These neoplasms originate from enterochromaffin-like cells of the gastric mucosa.

**PERSPECTIVES:**

Gastric neuroendocrine tumors (gNETs) are uncommon and heterogeneous, and require individualized management. In this largest Brazilian cohort, Type I gNET predominated, showing indolent behavior and favorable outcomes with endoscopic treatment. Type II tumors were rare and invariably linked to multiple endocrine neoplasia type 1, while Type III tumors presented aggressive features, advanced stage, and poor survival. Prognosis was strongly determined by tumor, lymph node, metastasis stage, highlighting the importance of accurate staging and histopathological evaluation.

## VISUAL ABSTRACT

**Figure:**
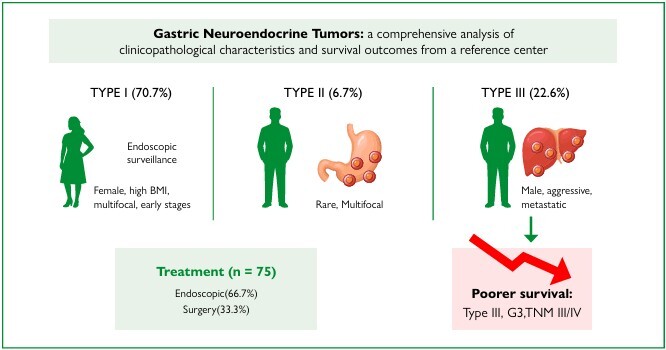

## RESUMO

**Racional:**

Tumores Neuroendócrinos Gástricos (TNEg) são neoplasias raras que surgem das células enterocromafins, representando um subconjunto distinto de neoplasias gástricas, com manejo clínico desafiador.

**Objetivos:**

Analisar a classificação, a indicação do tratamento e sobrevida de doentes com diagnóstico de TNEg.

**Métodos:**

Foram avaliados retrospectivamente pacientes diagnosticados com TNEg entre 2009 e 2025 em centro de referência no Brasil. Pacientes foram classificados de acordo com a WHO e critérios clinicopatológicos em Tipos I, II e III.

**Resultados:**

Dos 75 pacientes incluídos, 53 (70,7%) foram classificados como Tipo I, 5 (6,7%) como Tipo II e 17 (22,6%) como Tipo III. Ressecção cirúrgica foi realizada em 25 pacientes (33,3%), e endoscópica em 50 (66,7%). Tumores Tipo I tiveram associação com sexo feminino (p<0,001), multifocalidade (p<0,001), IMC elevado (p=0,002) e manejo endoscópico (p=0,008). Os tumores Tipo II foram raros e associados à MEN1, enquanto Tipo III foi predominante em homens, de alto grau (G3), e frequentemente metastáticos, exigindo ressecção cirúrgica e terapia sistêmica. Entre os 25 pacientes tratados cirurgicamente, foram principalmente do sexo masculino (52%) e incluía 12 pacientes (48,0%) com tumores Tipo I, 3 (12,0%) com Tipo II e 10 (40,0%) com tumores Tipo III. A análise de sobrevida mostrou resultados significativamente piores para os tumores Tipo III e G3. A análise multivariada identificou a idade avançada (HR 4,11; IC 95%: 1,14-14,80; p=0,030) e o estadio TNM III/IV (HR 5,42; IC 95%: 1,26-23,26; p=0,023) como preditores independentes de pior sobrevida.

**Conclusões:**

Os TNEg exibem um perfil clínico heterogêneo, com os tumores Tipo I predominando na população brasileira. O tipo do tumor, grau e estadiamento TNM definem manejo e prognóstico.

## INTRODUCTION

 Gastric cancer is estimated to be the fifth most common malignancy in Brazil between 2023 and 2025^16^. Adenocarcinoma represents the vast majority of gastric tumors, accounting for approximately 90% of cases, whereas other histological types include gastrointestinal stromal tumors, lymphomas, and gastric neuroendocrine neoplasms (NEN)^2^. The stomach is the most frequent site of gastrointestinal NENs, with an annual incidence of approximately 0.4 per 1,00,000 individuals^13,15,22^. According to the most recent World Health Organization Classification of Tumors of the Digestive System, NENs are divided into three major categories: gastric neuroendocrine tumors (gNETs), which are the most common, well-differentiated, and have any grade; neuroendocrine carcinomas (NECs), which are poorly differentiated and highgrade; and mixed neuroendocrine-non-neuroendocrine neoplasms (MiNENs), which are aggressive and harbor multiple molecular alterations. These neoplasms originate from enterochromaffin-like (ECL) cells of the gastric mucosa^1,10,11,14^. Diagnosis is usually established by endoscopic biopsy, with immunohistochemistry (IHC) confirming the neuroendocrine nature. IHC is typically positive for chromogranin A and synaptophysin. Determining the histological grade (G) is essential for prognosis. Gastric NETs are graded as G1, G2, or G3 according to mitotic count and proliferative index assessed by Ki-67. Importantly, well-differentiated G3 gNETs are distinguished from poorly differentiated NECs based on morphology and IHC features^1,5,10,13,19^. Gastric NETs are further subclassified into three main clinical and pathophysiological types, largely defined by their relationship with gastrin, associated conditions, and biological behavior. Type I tumors, the most common (70–80%), arise in the setting of autoimmune atrophic gastritis with hypergastrinemia and are typically indolent, with a metastatic risk of less than 5%. Type II tumors occur in the context of gastrinomas, most often associated with Zollinger-Ellison syndrome and multiple endocrine neoplasia type 1 (MEN1), and carry a slightly higher metastatic risk (around 7%). Type III tumors are sporadic, not associated with hypergastrinemia, and account for 15–25% of cases; they display aggressive biological behavior, often present with metastases, and are associated with poor long-term outcomes^5,10,11,13^. With advances in diagnostic modalities and the increasing availability of endoscopy in Brazil, a rise in gNET detection is expected. However, national data on the clinicopathological features and prognosis of gNETs remain scarce. The rarity and heterogeneous presentation of gNETs pose significant clinical challenges. gNETs require a multidisciplinary evaluation and an accurate risk stratification to determine appropriate management, which can range from simple endoscopic resection to gastrectomy with lymphadenectomy, depending on tumor type, grade, and stage^6,13^. Therefore, the present study aimed to evaluate the clinicopathological characteristics and survival outcomes of patients with gNET treated at a high-volume tertiary referral center in Brazil. 

## METHODS

 All patients with a histopathological diagnosis of gNET between 2009 and 2025 at our center were included in this study. Data were retrospectively collected and entered into a clinical database. Laboratory tests, imaging, endoscopic findings, and histopathological reports with IHC were reviewed. Patients with NEC and MiNEN were excluded. Clinical variables assessed included sex, age, body mass index (BMI), hemoglobin levels, gastrin levels, anti-parietal cell antibodies, American Society of Anesthesiologists (ASA) classification, and Charlson-Deyo comorbidity index (CCI), excluding age and gastric cancer. Postoperative complications were graded according to the Clavien-Dindo classification, with Clavien grade >II considered as major complications. Patients managed with endoscopic procedures (polypectomy, endoscopic mucosal resection, endoscopic submucosal dissection, and biopsy only) and those undergoing gastrectomy (wedge, total, or subtotal) were included. Indications and technical aspects of the procedures followed the recommendations of the Japanese Gastric Cancer Association^8^ and the Brazilian Gastric Cancer Association^2^. Pathological tumor, lymph node, metastasis (TNM) staging was defined according to the 9th edition of the American Joint Committee on Cancer staging system^3^. All surgical specimens were assessed in accordance with the College of American Pathologists protocols. Pathological variables included tumor size, tumor focality, invasion depth, presence of lymph node metastasis (N0 or N1), mitotic count, proliferative activity (Ki-67 index), IHC profile, and histologic grade (G). Grading was defined as follows: G1 (mitotic rate <2 per 2 mm^2^ and Ki-67 <3%); G2 (mitotic rate 2–20 per 2 mm^2^ or Ki-67 3–20%); and G3 (mitotic rate >20 per 2 mm^2^ or Ki-67 >20%). The primary tumor (pT) was staged as: T1 (mucosal or submucosal invasion and = 1 cm in greatest dimension); T2 (invasion of muscularis propria or >1 cm in greatest dimension); T3 (invasion through muscularis propria into subserosal tissue without serosal penetration); and T4 (invasion of visceral peritoneum/serosa or adjacent organs/structures)^2^. Patients who initially underwent diagnostic or therapeutic endoscopic procedures but subsequently required gastrectomy were analyzed in the surgical group. 

 Gastric NETs were subclassified into three clinical types based on clinicopathological features:

Type I: associated with atrophic gastritis, multiple polyps, and positive anti-parietal cell antibodies (when available);
Type II: associated with MEN1 or with evidence of a synchronous pancreatic or duodenal gastrinoma on imaging, biopsy, or pathology;
Type III: sporadic, isolated lesions without evidence of atrophic gastritis.

A brief summary of the characteristics of each type of gNET is presented in Table 1. 

**Table 1:** Neuroendocrine gastric tumors (gNETs) type.

Variables	Type I	Type II	Type III
Frequency	70–90%	7–15%	15–25%
Sex	Female (1:0.4)	No difference (1:1)	Male (2.8:1)
Associated features	Chronic autoimmune atrophic gastritis, with formation of autoantibodies targeting parietal cells	Zollinger-Ellison syndrome associated with gastrin producing tumors in the context of MEN1	Sporadic
Symptoms	Asymptomatic	Abdominal pain, GI bleeding related to ulcers, and secretory diarrhea	Abdominal pain, weight loss, and GI bleeding
Gastrin levels	High	Very high (>1000 pg/mL)	Low
Endoscopic features	Multiple small polyps or nodules in the gastric body or fundus (usually <1 cm)	Multiple tumors in the gastric body or fundus (usually <2 cm)	Single, large (usually >2 cm)Tumor
Risk of metastasis	Very low	Low	High (50% at diagnosis)
Prognosis	Excellent	Good	Poor

 Follow-up was performed every 3 months during the first year and every 6 months thereafter. The study was approved by the Ethics Committee of the Institution (plataformabrasil.saude.gov.br; CAAE: 84087624.5.0000.0068). 

### Statistical analysis

 Categorical variables were described as absolute values and percentages, whereas continuous variables were expressed as mean±standard deviation (SD) or as median with interquartile range (IQR), as appropriate. Categorical variables were compared using the ꭓ^2^ or Fisher’s exact test, and continuous variables using analysis of variance or the Kruskal-Wallis test. Overall survival (OS) was estimated by the Kaplan-Meier method and compared with the log-rank test. Survival was defined as the time from surgery or endoscopic treatment to death or last follow-up. Prognostic factors were assessed with the Cox proportional hazards model; variables with p<0.05 in univariable analysis were included in the multivariable model. Results are presented as hazard ratios (HRs) with 95% confidence intervals (CIs). All tests were two-sided, and p<0.05 was considered statistically significant. Analyses were conducted using Statistical Package for the Social Sciences (SPSS) version 20.0 (SPSS Inc., Chicago, IL). 

## RESULTS

 Of the 1,642 patients who underwent surgical or endoscopic treatment for gastric cancer at our institution, 75 (4.5%) were diagnosed with gNET and included in the analysis. The mean age was 56.2 years (SD±14.5), and most patients were female (61.2%). Treatment consisted of surgery in 25 patients (33.3%) and endoscopic resection in 50 patients (66.7%). Regarding clinicopathological types, 53 patients (70.7%) were classified as Type I, 5 (6.7%) as Type II, and 17 (22.6%) as Type III. Clinical characteristics according to gNET type are summarized in Table 2. 

**Table 2:** Clinical characteristics of patients according to gNET types. All patients (n=75).

Variables		Type I	Type II	Type III	p-value
		n=53 (%)	n=5 (%)	n=17(%)	
Sex					
	Female	42 (79.2)	2 (40)	2 (11.8)	<0.001
	Male	11 (20.8)	3 (60)	15 (88.2)	
Age (years)					
	Mean (SD)	65.1 (14.9)	47.1 (7.4)	59 (14.4)	0.280
Body mass index (kg/cm^2^)					
	Mean (SD)	29.8 (7.1)	22.5 (2.3)	24.5 (4.2)	0.002
Hemoglobin (g/dL)					
	Mean (SD)	13.1 (1.8)	13.1 (1.9)	10.5 (2.4)	<0.001
ASA					
	I/II	36 (69.2)	4 (80)	15 (88.2)	0.319
	III	16 (30.8)	1 (20)	2 (11.8)	
Comorbidity index (CCI)					
	CCI 0	37 (69.8)	4 (80)	12 (70.6)	0.266
	CCI 1	16 (30.2)	1 (20)	5 (29.4)	
Gastrin (pg/mL)					
	<100	5 (11.4)	0 (0)	0 (0)	1.000
	100–1,000	22 (50)	2 (50)	1 (100)	
	>1,000	17 (38.6)	2 (50)	0 (0)	
Atrophic gastritis					
	No	3 (5.7)	4 (80)	16 (94.1)	<0.001
	Yes	50 (94.3)	1 (20)	1 (5.9)	
Tumor location					
	Distal third	3 (5.7)	1 (20)	3 (17.6)	0.005
	Middle third	43 (81.1)	2 (40)	7 (41.2)	
	Proximal third	7 (13.2)	2 (40)	7 (41.2)	
Multifocality					
	No	9 (17.0)	2 (40)	17 (100)	<0.001
	Yes	44 (83.0)	3 (60)	0 (0)	
PET-CT*					
	No	50 (94.3)	3 (60)	15 (88.2)	0.058
	Yes	3 (5.7)	2 (40)	2 (11.8)	

SD: standard deviation; CCI: Comorbidity index.

* Gallium-68, Fluorodeoxyglucose or Octreoscan.

 Female sex, presence of atrophic gastritis, multifocal tumors, and higher BMI were significantly associated with Type I tumors. Endoscopic ultrasonography was performed in nine patients: seven with Type I (four with mucosal and three with submucosal invasion) and two with Type II (one with mucosal and one with muscular invasion). Anti-parietal cell antibodies were tested in 29 patients with Type I tumors and were positive in 15 (51.7%). All five patients with Type II tumors had MEN1. 

 Pathological and treatment-related characteristics are summarized in Table 3. Patients with Type I tumors were predominantly managed with endoscopic resection, whereas Types II and III were more often treated surgically. Type I gNETs were predominantly histologic grade G1, whereas Type III tumors were more frequently classified as G3, showed higher Ki-67 index values, and were diagnosed at more advanced stages, often with metastatic disease. Among the 17 patients with Type III gNETs, 9 (52.9%) received palliative chemotherapy, and 5 (29.4%) underwent radiotherapy. None of the patients with Type I or Type II tumors required adjuvant or palliative systemic therapy. 

**Table 3:** Pathological and treatment characteristics of patients according to gNET types. All patients (n=75).

Variables		Type I	Type II	Type III	p-value
		n=53 (%)	n=5 (%)	n=17(%)	
Type of treatment					
	Gastrectomy	12 (22.6)	3 (60)	10 (58.9)	0.008
	Endoscopic resection/biopsy	41 (77.4)	2 (40)	7 (41.2)	
Immunohistochemical markers					
	Cromogranin A	51 (96.2)	5 (100)	16 (94.1)	-
	Synaptophysin	50 (94.3)	5 (100)	16 (94.1)	
Ki-67 index (%)					
	<3	33 (62.3)	2 (40)	0 (0)	<0.001
	3–20	20 (37.7)	2 (40)	4 (23.5)	
	>20	0 (0)	1 (20)	13 (76.5)	
Number of mitoses (per 10 GPF)					
	0–1	51 (96.2)	4 (80)	12 (70.6)	0.008
	2–19	2 (3.8)	1 (20)	2 (11.8)	
	>20	0 (0)	0 (0)	3 (17.6)	
Histologic grade (G)					
	G1	33 (62.3)	2 (40)	0 (0)	<0.001
	G2	20 (37.7)	2 (40)	4 (23.5)	
	G3	0 (0)	1 (20)	13 (76.5)	
Tumor size (cm)					
	Mean (SD)	0.7 (0.8)	0.8 (1.1)	4.7 (2.5)	<0.001
T					
	T1/T2	52 (98.1)	5 (100)	5 (29.4)	<0.001
	T3/T4	1 (1.9)	0 (0)	12 (70.6)	
N					
	NX/N0	46 (86.8)	3 (60)	8 (47.1)	0.003
	N+	7 (13.2)	2 (40)	9 (52.9)	
M					
	M0	53 (100)	5 (100)	8 (47.1)	<0.001
	M1	0 (0)	0 (0)	9 (52.9)	
TNM					
	I/II	46 (86.8)	3 (60)	3 (17.6)	<0.001
	III/IV	7 (13.2)	2 (40)	14 (82.4)	

SD: standard deviation; TNM: tumor, lymph node, metastasis

 Among the 25 surgically treated patients, most were men (52%), with a mean age of 52 years. Distribution by gNET types included 12 patients (48.0%) with Type I, 3 (12.0%) with Type II, and 10 (40.0%) with Type III tumors. Gastrectomy with D2 lymphadenectomy was performed in the majority (76.0%), and open surgery (80%) was more common than minimally invasive approaches (20%). Only two patients underwent palliative resections due to metastatic disease. 

 Regarding histologic grade, most tumors were classified as G2 (44%), followed by G3 (32%). Multifocal disease was observed in 32% of cases. In terms of stage distribution, three patients (12.0%) were stage I, 6 (24.0%) stage II, 14 (56.0%) stage III, and 2 (8.0%) stage IV. The median postoperative hospital stay was 12 days (IQR 8–15), and five patients (20%) experienced major postoperative complications. Four patients received neoadjuvant therapy, and four received adjuvant chemotherapy. Detailed clinicopathological data are summarized in Table 4. 

**Table 4:** Clinicopathological characteristics of patients with gNET undergoing gastrectomy.

Variables		n
Sex		
	Female	12 (48)
	Male	13 (52)
gNET types		
	I	12 (48)
	II	3 (12)
	III	10 (40)
Surgical technique		
	Open	20 (80)
	Minimally invasive	5 (20)
Ressection type		
	Wedge	1 (4)
	Subtotal	9 (36)
	Total	15 (60)
Lymphadenectomy		
	D0	1 (4)
	D1	5 (20)
	D2	19 (76)
Number of lymph nodes		
	Mean (SD)	37 (17)
Complications – Clavien-Dindo Score		
	None/Clavien I–II	20 (80)
	Clavien III–IV	5 (20)
Length of stay (days)		
	Median (IQR)	12 (8–15)

SD: standard deviation; IQR: interquartile range.

**Figure 1:**
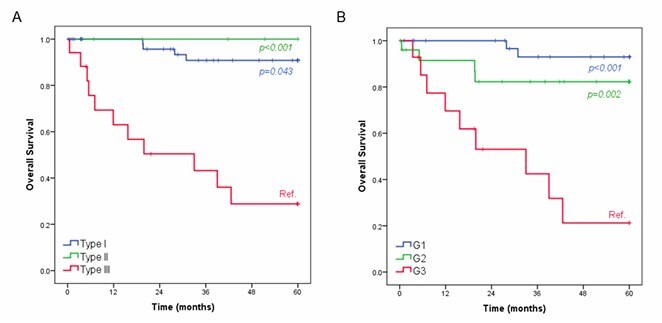
Overall survival according to gNET types (A) and histological grade (B).

 The median follow-up for the entire cohort was 49.9 months (IQR 19.7–60), with an estimated 5-year OS of 76.1%. At the last follow-up, 15 patients had died. OS significantly differed according to gNET types (Figure 1A). Patients with Type III tumors had worse outcomes compared with those with Type I (p<0.001) and Type II (p=0.043). The median OS for Type II tumors was 33 months. No significant difference was observed between Types I and II gNETs (p=0.593). 

 When analyzed by histologic grade, G3 tumors had the poorest prognosis, with a median OS of 33 months, significantly shorter than that of G1 (p<0.001) and G2 tumors (p=0.002). No significant survival difference was observed between G1 and G2 tumors (p=0.157) (Figure 1B). 

 In multivariable analysis (Table 5), advanced age (HR 4.11; 95%CI 1.14–14.80; p=0.030) and TNM stage III/IV (HR=5.42; 95%CI: 1.26–23.26; p=0.023) were independently associated with worse OS. 

**Table 5:** Univariable and multivariable analysis for overall survival.

Variables	Univariable		p-value	Multivariable		p-value
	HR	95%CI		HR	95%CI	
Male (vs. female)	5.12	1.63–16.11	0.005	1.25	0.33–4.80	0.744
Age =60 (vs. <60 years)	3.17	1.01–9.97	0.048	4.11	1.14–14.80	0.030
ASA III (vs. ASA I/II)	1.84	0.65–5.17	0.250	-	-	-
Gastrectomy (vs. ESD)	1.25	0.44–3.51	0.673	-	-	-
Grade 3 (vs Grades 1 and 2)	9.72	3.41–27.69	<0.001	5.46	0.80–37.11	0.082
TNM III/IV (vs I/II)	8.53	2.71–26.87	<0.001	5.42	1.26–23.26	0.023
Type III (vs Type I/II)	13.55	4.29–42.80	<0.001	0.74	0.14–3.98	0.729

HR: hazard ratio; CI: confidence interval; ASA: American Society of Anesthesiologists; ESD: endoscopic submucosal dissection; TNM: Tumor, Lymph node, Metastasis.

## DISCUSSION

 In the present study, we retrospectively evaluated 75 patients with gNETs, corresponding to 4.5% of all patients treated for gastric neoplasms at our institution. At first impression, this rate appears higher than expected. This may be explained by the referral nature of our tertiary center, which frequently receives complex cases from other hospitals. Moreover, the relative unfamiliarity with gNETs in general practice may lead to a greater tendency to refer these patients. The presence of multidisciplinary teams, including endocrinology and immunology specialists, may also increase the likelihood of identifying patients with autoimmune disorders or MEN-1 syndrome, contributing to the higher prevalence observed. As reported in large series, the majority of gNETs were Type I, characterized by multiple and small polyps, female predominance, low comorbidity burden, and early-stage disease. Our findings are consistent with Asian cohorts, which describe indolent behavior and very low metastatic risk, supporting endoscopic resection and surveillance as safe strategies^4,12^. 

 Type I gNETs are typically associated with autoimmune atrophic gastritis and may coexist with other autoimmune disorders, such as thyroiditis, type 1 diabetes, vitiligo, and celiac disease^17^. Unfortunately, due to the retrospective nature of this study, the presence of such comorbidities could not be systematically assessed, although clinicians should remain vigilant for these associations. Anti-parietal cell antibodies, tested in a subgroup of type I patients, were positive in more than half of the cases, reinforcing their potential role as a useful adjunct marker in clinical practice. Interestingly, patients with Type I tumors displayed higher BMI. This may be explained by the greater symptom burden in Type II, related to gastrinoma, and in Type III, which are often diagnosed at more advanced stages, though this hypothesis could not be formally tested. 

 Type II gNETs were rare in our cohort, as described in the literature. The small number of cases may also reflect classification bias, as patients with gastrinomas might have been categorized as pancreatic tumors and treated accordingly, thus not meeting the criteria for inclusion in the present study, even when associated gastric lesions were present. All Type II patients had MEN-1, with multifocal polyps in early disease stages, similar to Type I. Their management should prioritize surgical resection of the gastrinoma, while gastric lesions can usually be managed endoscopically, in the context of multidisciplinary care^5,13^. 

 In contrast, Type III tumors were more frequent in males, solitary, larger in size, and diagnosed at advanced stages, often with lymph node or distant metastases. These patients had significantly worse survival, confirming their aggressive biology. All patients who required systemic therapy, including chemotherapy or radiotherapy, belonged to this group. Although Type III tumors were strongly associated with poor outcomes in univariable analysis, this effect was not retained in the multivariable model, where advanced age and TNM stage were the only independent predictors of mortality. This finding suggests that prognosis in gNET is largely determined by stage at diagnosis, rather than histological type per se, emphasizing the importance of early detection^20,21^. Current evidence supports platinum-based chemotherapy in metastatic gNET, but outcomes remain poor, with median survival of approximately 11 months. Neoadjuvant therapy may be considered in selected patients, though evidence is limited^18,21^. The lack of effective systemic therapy remains one of the greatest challenges in this subset. 

 The diagnostic work-up of gNETs can be complex. Gastrin levels are typically associated with Type I and II tumors; however, one-third of our patients did not have gastrin measured at diagnosis, limiting subgroup analysis. Even among Type I patients, gastrin levels were heterogeneous, ranging from normal to markedly elevated (>1,000 pg/mL), which can mimic gastrinoma. Similarly, about half of Type II patients had intermediate gastrin levels, illustrating the limited accuracy of gastrin alone to distinguish tumor types. Atrophic gastritis and multifocality were highly associated with Type I tumors but were not exclusive, as one patient with Type II and another with Type III also presented with atrophic gastritis. Conversely, Type III tumors were invariably solitary, reinforcing their distinct biology. 

 Regarding treatment, endoscopic management predominated in Type I, consistent with guidelines^6,13^. Some patients with Type III tumors were classified as having undergone endoscopic treatment, but in reality, these were diagnostic biopsies in metastatic patients, reflecting their poor prognosis. Surgical treatment was mainly reserved for Type II and III tumors, most often through gastrectomy with D2 lymphadenectomy. Although open surgery was predominant, minimally invasive approaches have been progressively adopted in recent years and should be considered feasible alternatives^7,9^. In selected patients, wedge resection with lymph node sampling was performed, avoiding extended gastrectomy and allowing better postoperative outcomes, in accordance with international guidelines^6^. 

 Histological grade clearly correlated with outcomes, with G3 tumors showing dismal survival compared to G1 and G2. In multivariable analysis, TNM stage remained the strongest independent prognostic factor, in line with its well-established role in gastric malignancies. Grade 2 tumors remain the most challenging group, as they can present with variable biological behavior across all types. It is worth noting that poorly quantified Ki-67, poorly representative biopsies, or small samples may underestimate tumor grade. This requires accurate risk stratification and multidisciplinary evaluation to determine appropriate treatment, which is not always straightforward with available tests (Ki-67, histological grade, and imaging). 

 This study has limitations, particularly related to its retrospective design and incomplete availability of biomarkers such as gastrin and anti-parietal cell antibodies. Advanced diagnostic tools such as Gallium-68 PET/CT (Positron Emission Tomography/Computed Tomography) were not routinely available, which could have improved tumor staging and detection of gastrinomas in MEN-1 patients^13,21^. Despite these limitations, this series represents the largest Brazilian cohort of patients with gNETs, highlighting the predominance of Type I tumors and the aggressive course of Type III. Taken together, our findings underscore the heterogeneity of gNETs and the importance of early diagnosis, careful histopathological assessment, and individualized treatment strategies guided by tumor type, grade, and stage. 

## CONCLUSIONS

 Gastric NETs are uncommon and heterogeneous, and require individualized management. In this largest Brazilian cohort, Type I gNET predominated, showing indolent behavior and favorable outcomes with endoscopic treatment. Type II tumors were rare and invariably linked to MEN-1, while Type III presented aggressive features, advanced stage, and poor survival. Prognosis was strongly determined by TNM stage, highlighting the importance of accurate staging and histopathological evaluation. 

## Data Availability

The datasets generated and/or analyzed during the current study are available from the corresponding author upon reasonable request.
